# Case Report: A case of a giant right ventricular wall hematoma caused by coronary artery perforation during percutaneous coronary intervention

**DOI:** 10.3389/fcvm.2026.1877014

**Published:** 2026-06-23

**Authors:** Yue Bao, Jun Ma, Lei Li

**Affiliations:** Department of Cardiology, Wuhan Asia Heart Hospital, Wuhan, China

**Keywords:** cardiac tamponade, coronary artery bypass grafting, coronary artery perforation, percutaneous coronary intervention, right ventricular wall hematoma

## Abstract

Iatrogenic coronary artery perforation (CAP) is a rare but critical complication of percutaneous coronary intervention (PCI). While Ellis type III CAP typically causes massive pericardial effusion and acute cardiac tamponade, herein, we report an atypical case of type III coronary artery perforation in a 71-year-old male patient during PCI. The patient presented with hemodynamic instability mimicking cardiac tamponade. Imaging revealed a subepicardial hematoma on the right ventricular surface with only a small amount of pericardial effusion. The patient was successfully rescued by emergency coronary artery bypass grafting combined with hematoma evacuation.

## Background

Coronary artery perforation (CAP) is a devastating procedural complication of percutaneous coronary intervention (PCI), occurring in 0.1%–0.8% of cases. The Ellis type III perforation poses the highest clinical risk, often rapidly progressing to acute cardiac tamponade and cardiogenic shock, with an in-hospital mortality rate ranging from 19% to 27.2% (1–3). Type III perforation is typically characterized by free extravasation of blood into the pericardial cavity, resulting in massive pericardial effusion and cardiac tamponade (4, 5). However, postperforation bleeding can rarely be localized beneath the epicardium, forming a subepicardial hematoma with only minimal pericardial effusion—a condition prone to misdiagnosis (6, 7). In this study, we report a rare case of a patient with compressive subepicardial hematoma on the right ventricular wall secondary to an Ellis type III coronary artery perforation who was successfully treated with emergency coronary artery bypass grafting (CABG) and hematoma evacuation. This case enriches the clinical experience in the management of such atypical and critical patients.

## Case presentation

A 71-year-old man was admitted to the hospital with a half-month history of chest tightness. He had a history of hyperlipidemia and was a long-term smoker. His physical examination on admission revealed no abnormalities. Laboratory tests revealed a high-sensitivity cardiac troponin I (hs-cTnI) level of 0.0952 ng/mL (reference range: 0–0.02 ng/mL). An electrocardiogram (ECG) showed inverted T waves in the inferior leads. Echocardiography demonstrated left atrial enlargement, decreased left ventricular diastolic function, and no pericardial effusion. The admission diagnosis was acute non-ST-segment elevation myocardial infarction. Oral aspirin and clopidogrel were administered preoperatively. With the patient's informed consent, coronary angiography (CAG) was performed at 10:40 on 8 March 2026.

**Figure 1:**
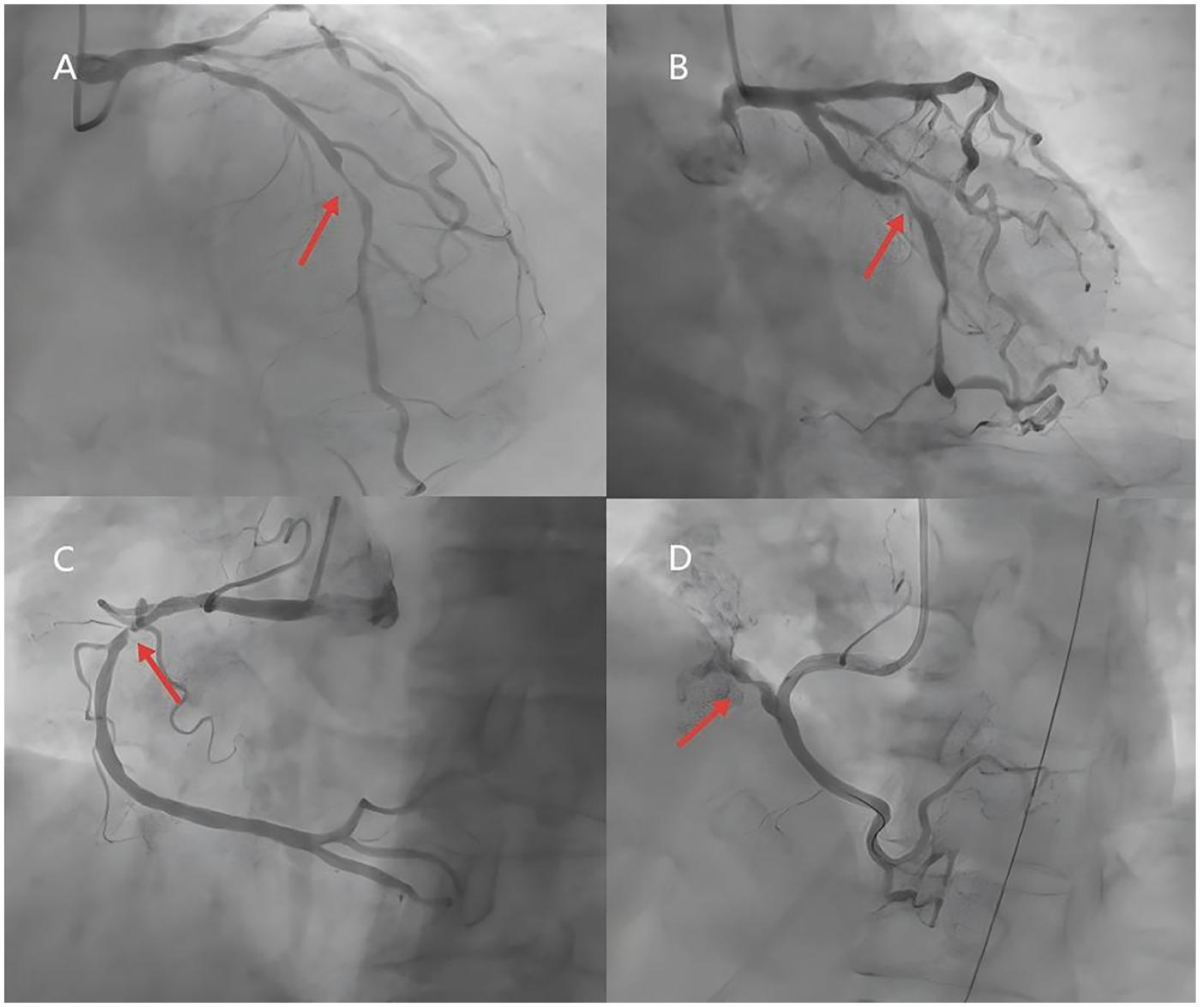
**(A–D)** Coronary angiography shows **(A)** severe stenosis in the mid-LAD (arrow); **(B)** severe stenosis in the proximal LCX (arrow); **(C)** severe stenosis in the mid-RCA (arrow); and **(D)** contrast medium extravasation in the mid-RCA (arrow).

**Figure 2:**
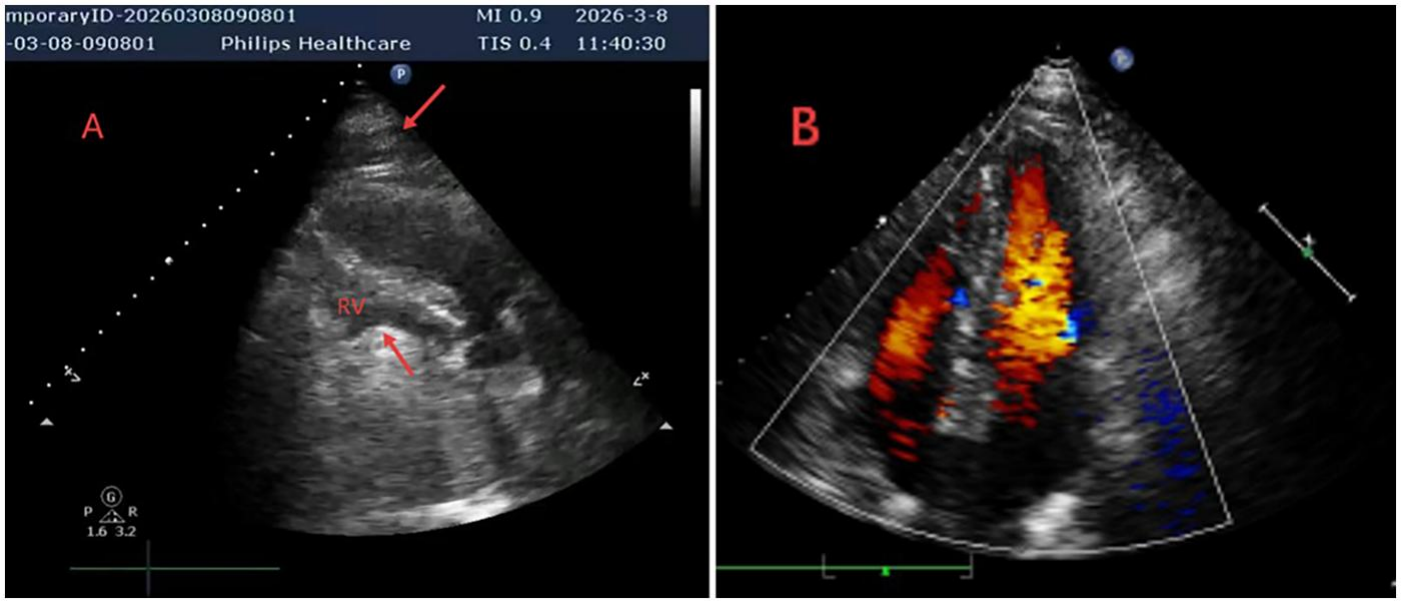
**(A)** Echocardiography shows a large right ventricular hematoma (arrow) with mild pericardial effusion (arrow). **(B)** Echocardiography shows resolution of the hematoma, with the right atrium and right ventricle returning to normal size.

**Figure 3:**
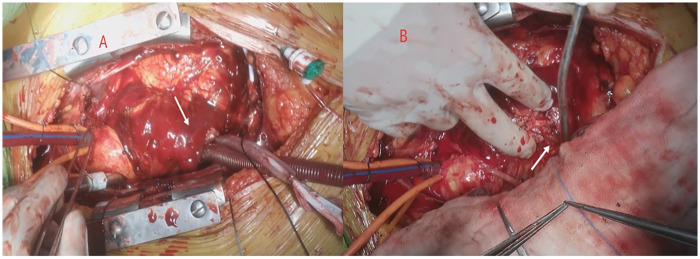
**(A)** Thoracotomy: A subepicardial hematoma was present beneath the right ventricular epicardium after pericardial opening (arrow). **(B)** Thoracotomy: After dissection of the RCA, blood spurted from the mid-segment, suggesting RCA perforation (arrow).

**Figure 4:**
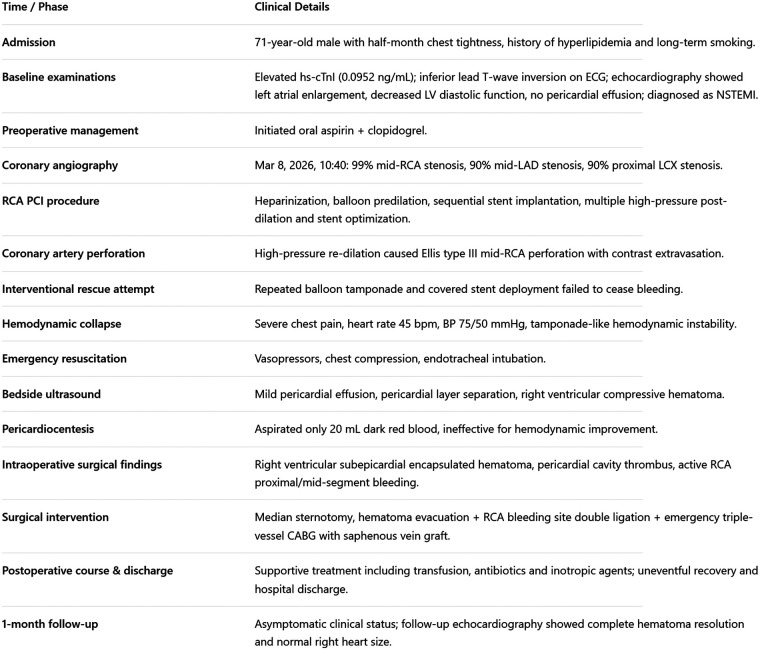
CARE-style timeline.

Percutaneous coronary intervention (PCI procedure: CAG revealed 99% mid-RCA stenosis, 90% mid-LAD stenosis, and 90% proximal left circumflex artery (LCX) stenosis (Figures 1A–C). Because of the severe and unstable right coronary artery (RCA) lesion, urgent PCI of the RCA was planned. Intraoperatively, 6,500 units of heparin were administered, and the activated clotting time was maintained at approximately 300 s. Balloon 1 (PN20020, Batai, Zhejiang) was advanced along the guidewire to the mid-RCA lesion, and predilation was performed at 8–10 atm for 6–7 s. Stent 1 (FBP3033, MicroPort, Shanghai), Stent 2 (FBP3033, MicroPort, Shanghai), and Stent 3 (RSINT 4.0 mm × 22 mm, Medtronic) were then delivered over the guidewire to the RCA lesion and implanted sequentially, and postdilation was performed at 9–10 atm for 7–8 s. Balloon 2 (PG35012, Batai, Zhejiang), Balloon 3 (Quantum 3.75 mm × 12 mm, Boston Scientific), and Balloon 4 (833-4012, Biomime, Guangdong) were sequentially advanced into the RCA stents over the guidewire for stent optimization, and dilation was performed at 12–16 atm for 7–8 s, 16–18 atm for 8 s, and 16–20 atm for 6–8 s, respectively. A repeat angiography revealed incomplete stent expansion in the mid-RCA. Balloon 3 was repositioned within the mid-RCA stent, and high-pressure dilation was applied at 18–24 atm for 5–7 s. A subsequent angiography demonstrated contrast extravasation at the mid-RCA, suggesting coronary artery rupture (Figure 1D). Balloon 5 (PG35012, Batai, Zhejiang) was immediately advanced to the mid-RCA stent and inflated at 10–12 atm to seal the rupture. A covered stent (FBP3518, MicroPort, Shanghai) was then delivered along the guidewire to the mid-RCA and deployed at 9–10 atm for 7–8 s. A follow-up angiography, however, showed persistent massive contrast extravasation in the mid-RCA. Balloon 6 (Quantum 3.0 mm × 12 mm, Boston Scientific) was placed inside the mid-RCA stent and inflated at 16 atm for hemostasis, but the bleeding was not controlled. The patient rapidly developed severe chest pain, accompanied by a drop in heart rate to 45 beats per minute and blood pressure to 75/50 mmHg, with hemodynamic collapse mimicking acute cardiac tamponade. Vasopressors, chest compressions, and endotracheal intubation were administered. Pericardiocentesis was performed immediately, aspirating approximately 20 mL of dark red blood. A bedside ultrasound showed separation of the visceral and parietal layers of the pericardial cavity with echo-free spaces distributed between them, indicating a small amount of pericardial effusion (Figure 2A). The surgical department was consulted for emergency CABG combined with hematoma evacuation. A median sternotomy was performed. Blood clots were found on the right atrial and right ventricular surfaces of the pericardial cavity, along with a subepicardial hematoma on the right ventricular surface (Figure 3A). Active bleeding was noted in the proximal and midsegments of the RCA (Figure 3B). The pericardial cavity was cleared of blood clots. The proximal and midsegments of the RCA were exposed, the bleeding site was identified, and double ligation was performed for hemostasis. Given the patient’s severe stenosis in the RCA, left anterior descending artery (LAD), and LCX, coronary artery bypass grafting (CABG) was performed for all three vessels. The great saphenous vein was harvested, and a saphenous vein graft was sequentially anastomosed to the posterior descending artery and the LAD. An end-to-side anastomosis was created between the obtuse marginal branch vein graft and the LAD vein graft. No bleeding leakage was detected on inspection. The operation was successful. Postoperatively, the patient received blood transfusions, antibiotic therapy, inotropic support, metabolic stabilization, and anti-ischemic medications. The patient was discharged in an improved condition and was put on oral aspirin, clopidogrel, and rosuvastatin after discharge. At the 1-month follow-up, the patient was asymptomatic. Echocardiography revealed no hematoma, and the right heart chambers returned to normal size (Figure 2B). A detailed CARE-compliant patient timeline is provided in Figure 4.

## Discussion

CAP is a severe, acute, and life-threatening complication that occurs during PCI. Currently, the Ellis classification is widely used in clinical practice and comprises three types (1): Type I is characterized by a contained extraluminal crater in the vascular wall without contrast medium extravasation; type II presents with epicardial or intramyocardial contrast medium staining without free extravasation; and type III is defined as a vascular rupture with a diameter >1 mm, accompanied by persistent free extravasation of contrast medium, which may be complicated by cardiac tamponade in severe cases. Large-scale clinical studies have shown that Ellis type III perforations account for 37.2%–44% of all coronary artery perforations, and it is also the primary high-risk subtype associated with acute cardiac tamponade, cardiogenic shock, and in-hospital mortality (2, 3). Type III CAP typically manifests as sudden chest pain, bradycardia, and refractory hypotension during the procedure. Echocardiography typically demonstrates a significant pericardial effusion and right ventricular diastolic collapse. Timely pericardiocentesis usually results in rapid hemodynamic improvement (4, 5). Relevant guidelines recommend a stepwise intervention strategy: First-line management involves temporary occlusion of the rupture with a low-pressure balloon; early implantation of covered stents is recommended for main coronary artery perforations; immediate pericardiocentesis is indicated for patients with acute cardiac tamponade; if interventional hemostasis is ineffective and hemodynamics are difficult to maintain, emergency surgical conversion is required, including surgical repair or ligation of the bleeding site combined with coronary artery bypass grafting (5–8). International multicenter data show that for Ellis type III perforation, the utilization rate of pericardiocentesis is 81.2%, the implantation rate of covered stents is 48.3%, the emergency CABG rate is 17.2%, and the overall in-hospital mortality rate is 18.1% (2). Registry data from more than 520,000 PCI procedures in the United Kingdom show that the rate of emergency surgery in patients with type III perforation is 39%, with an associated mortality rate of approximately 19% (3). To date, most studies have focused on typical cardiac tamponade caused by massive pericardial effusion, while case reports of encapsulated subepicardial hematomas with compression of cardiac chambers after CAP are rare (6, 7). In contrast, atypical patients present with chest pain, bradycardia, and refractory hypotension that closely mimic acute cardiac tamponade. In such patients, bleeding tends to be confined to the subepicardial space, forming a localized hematoma that compresses the right ventricle, rather than spreading freely into the pericardial cavity, thus resulting in only minimal pericardial effusion. Conventional pericardiocentesis, which alleviates cardiac compression by draining free pericardial fluid, is ineffective for hematoma-induced mechanical obstruction. Overreliance on this procedure can mislead clinical judgment, delay definitive surgical intervention, and cause serious adverse outcomes (9, 10). Most reported subepicardial hematomas predominantly lead to left atrial compression, and only one case has been documented with right ventricular hematoma related to Ellis type II perforations (11–13).

The present report describes a rare case of Ellis type III perforation complicated by a right ventricular subepicardial hematoma with marked ventricular cavity compression. Intraoperatively, Ellis type III perforation of the mid-RCA was confirmed. The patient rapidly developed tamponade-like symptoms; meanwhile, a bedside ultrasound revealed only mild pericardial effusion, pericardial layer separation, and localized echo-free areas. Repeated pericardiocentesis yielded limited efficacy. Thoracotomy confirmed that the perforation bleeding was confined beneath the epicardium, forming an encapsulated hematoma directly compressing the right heart chambers, representing an atypical variant of type III perforation (6, 7). The pathogenesis may likely involve several factors: The perforation occurred in the epicardial course of the mid-segment of the RCA. Intraoperative operations such as balloon occlusion and attempted stent implantation may have changed the direction of bleeding and slowed the bleeding speed, promoting the formation of a localized hematoma (9); the right ventricular wall is thin, with poor diastolic compliance and low tolerance to external mechanical compression. Consequently, even a small hematoma can significantly hinder ventricular diastolic filling (10). For PCI patients with sudden hemodynamic instability and high suspicion of CAP, atypical subepicardial hematoma should be considered when typical symptoms are accompanied by minimal pericardial effusion and ineffective pericardiocentesis. Early echocardiography is crucial for definitive diagnosis and timely therapeutic adjustment. It can clearly identify the location of the hematoma, its size, and the degree of right ventricular compression and distinguish typical tamponade secondary to massive pericardial effusion from atypical compression caused by subepicardial hematoma (9, 10, 14). For patients with active bleeding and persistent cardiac compression, interventional therapy alone cannot relieve mechanical obstruction. Early surgical evacuation of the hematoma, ligation of the bleeding vessel, and concurrent CABG are critical for salvaging patients (2–4). Furthermore, timely multidisciplinary collaboration among interventional cardiology, echocardiography, anesthesiology, and cardiac surgery shortens the time to definitive treatment, avoids diagnostic delays, and improves survival in such critically ill patients with atypical CAP (8). This study is a single-center retrospective case report with a limited sample size, which limits our ability to systematically elucidate the pathogenesis. Further studies with larger cohorts are warranted to validate the findings of this study.

## Data Availability

The original contributions presented in the study are included in the article/Supplementary Material, and further inquiries can be directed to the corresponding author.
